# Impact of COVID-19 lockdown on methadone and buprenorphine prescriptions in England primary cares: an interrupted time series analysis

**DOI:** 10.1186/s12954-025-01354-1

**Published:** 2025-11-25

**Authors:** Yi-Chen Chang, Wan-Chuen Liao, Li-Chia Chen, Teng-Chou Chen

**Affiliations:** 1 Department of Pharmacy, National Yang-Ming Chiao-Tung University, Number 155, Section 2 Linong Street, Beitou District, Taipei City, 112304 Taiwan; 2 Centre for Pharmacoepidemiology and Drug Safety, Division of Pharmacy and Optometry, The University of Manchester, 1st Floor, Stopford Building, Oxford Road, Manchester, M13 9PT UK; 3 School of Dentistry, College of Medicine, National Taiwan University, Number 1, Changde Street, Zhongzheng District, Taipei City, 100 Taiwan

**Keywords:** Opioid-related disorders, COVID-19, Socioeconomic factors, Methadone, Buprenorphine, England

## Abstract

**Background:**

The COVID-19 lockdown prompted changes in accessing opioid agonist therapy in England, but the lockdown and further adaptations could exacerbate inequalities in opioid agonist therapy availability across practices with different socioeconomic statuses. This study aimed to evaluate the impact of the COVID-19 lockdown on the prescribing of methadone and buprenorphine and how general practices located within areas with differing socioeconomic status responded to these policies in England.

**Methods:**

This quasi-experimental study used a health administrative practice-level dispensing database from March 2019 to February 2022 and socioeconomic deprivation from the Office for National Statistics in England. General practices that prescribed methadone or buprenorphine were included. The monthly number of Defined Daily Doses (DDDs) and dispensed items were quantified. The monthly changes in DDDs and dispensed items during the COVID-19 lockdown were also calculated. Interrupted time series analysis was used to evaluate the impact of the COVID-19 lockdown in March 2020. For practices with consistent prescribing (any OAT prescribing from December 2019 to February 2020 and 6 months during the lockdown), a group-based trajectory model explored the variability between practices.

**Results:**

A significant level elevation (β_2_ = 473,678.3, p = 0.002) and slope decrease (β_3_ = − 46,396.6, p = 0.03) in DDDs of methadone, and a level elevation (β_2_ = 114,041.9, p = 0.002) in DDDs of buprenorphine were found after COVID-19 lockdown. Practices located in the more deprived areas were more likely to prescribe OAT consistently, and 16.8% of practices were categorised into decreasing methadone dispensing during the COVID-19 lockdown, and 5.4% of practices were classified as increasing dispensing of buprenorphine. There was a tendency for practices located in deprived areas to be grouped into the trajectory of decreasing methadone dispensing, but not with buprenorphine.

**Conclusions:**

The COVID-19 lockdown limited the prescribing of methadone and increased the prescribing of buprenorphine in England. Further studies should adopt individual patient data to investigate the potential reasons for limiting the prescribing of methadone.

**Supplementary Information:**

The online version contains supplementary material available at 10.1186/s12954-025-01354-1.

## Background

Opioid use disorder (OUD) affects over 250,000 people in the United Kingdom (UK), with opioid-related deaths reaching around 2500 deaths in 2023 [1, 2]. Opioid agonist therapy (OAT) with either methadone or buprenorphine can effectively reduce opioid misuse and all-cause mortality for patients with OUD [3, 4]. Supervised methadone consumption is recommended by the guidance to reduce the risk of overdose, and sudden discontinuation of methadone or buprenorphine is not advisable due to the potential for withdrawal symptoms that could lead to illicit drug use and overdose [5].

OAT is prescribed at general practices and dispensed in community pharmacies in England. Methadone was recommended to be prescribed with supervision from treatment initiation until the patient achieves stability, and physicians were advised to consider unsupervised take-away doses when prescribing buprenorphine before the Coronavirus disease 2019 (COVID-19) pandemic [6].

The COVID-19 pandemic placed unprecedented strain on healthcare systems globally, prompting the UK to implement a national lockdown to reduce the burden on the healthcare system [7, 8]. In response to the lockdown policy, the use of telemedicine was increased, and adaptations were made to the supply of OAT services in general practice. Methadone could be prescribed for one to two weeks 'take-home doses', and patients were allowed to self-administer without supervision to reduce in-person contact [9]. Similarly, buprenorphine remained to be prescribed for unsupervised consumption during the COVID-19 pandemic (Supplemental Material Table S1) [9].

A patient-centered approach is central to the management of opioid use disorder [6]. Unsupervised consumption can offer benefits, including increased autonomy, reduced travel burden, and lower stigma [10, 11]. These advantages may help patients maintain employment, pursue education, and enhance their psychosocial stability [10, 11]. While the COVID-19 lockdown may have limited access to these benefits, they remain essential considerations in treatment planning. However, the rapid shift of methadone to unsupervised consumption also raised concerns regarding the risk of overdose and misuse, treatment dropout, the inability to assess the patient's status accurately, and the loss of benefits of supervised consumption to patients and healthcare providers [12, 13].

COVID-19 lockdown and the adaptations may have further exacerbated inequities in OAT access. Socioeconomic status (SES) is a known determinant of health outcomes, with individuals in more deprived areas experiencing higher rates of OUD, drug-related deaths, and greater barriers to healthcare access [14–16]. Video-based consultations were more accessible to patients with higher SES during the COVID-19 lockdown in the United States [17]. The evaluation of patient stability for methadone involved multiple-dimensional assessments, including adherence, psychosocial function, risk of abuse, and safety storage [6]. Limited access to video-based consultations may hinder healthcare providers’ ability to assess patient stability [17], which could in turn restrict equitable access to methadone treatment [16]. In addition, deprived SES was associated with barriers to housing, and patients with deprived SES might be reluctant to receive ‘take-home doses’ due to the concern about securely storing methadone [18], [19].

While a prior study was conducted on limited data from selected regions in Scotland to observe the early impact of COVID-19 and found a declining trend in the dispensing of methadone [20], no national-level analysis has quantified the impact of COVID-19 lockdown policies on OAT prescribing in England, nor has there been a comprehensive evaluation of socioeconomic disparities in response to these policies. To inform future OAT guidelines and promote equitable access to OAT in primary care, this study aims to quantify the impact of the COVID-19 lockdown measures on the prescribing of methadone and buprenorphine in English general practices and to assess how socioeconomic factors influenced the variability in response to these changes.

## Methods

### Study design, data sources, and study population

This quasi-experimental study examined the impacts of the COVID-19 lockdown (started from March 2020 and ended in February 2021) on prescription OAT dispensed patterns in England, utilising publicly available aggregate-level data, English Prescribing Data (EPD), from March 2019 to February 2022 from the National Health Service Business Services Authority [21].

The EPD is an administrative database that includes prescriptions issued by general practitioners and dispensed in community pharmacies in England. Medicine name, quantity, and number of items (medicine appears on the prescription form) of each prescribed and dispensed medicine were collected monthly and presented by practices. The monthly practice-level prescription data of methadone and buprenorphine were retrieved from the EPD. The socioeconomic information was sourced from the 2019 Index of Multiple Deprivation (IMD) by the Department for Levelling Up, Housing and Communities [19].

This study included all general practices in England that had methadone or buprenorphine prescription dispensed for opioid dependence during the study period. According to the British National Formulary (BNF), medications for opioid dependence were identified by specific codes, including methadone (BNF code: 0410030C0), buprenorphine/naloxone (BNF code: 0410030B0), and buprenorphine (BNF code: 0410030A0).

### Outcome measures

The primary outcomes were the monthly number of (1) Defined Daily Doses (DDDs) and (2) dispensed items (i.e., the number of medicines listed on the prescription form) for methadone and buprenorphine. The monthly number of DDDs provides a standardised measure of the total volume of medication dispensed. In contrast, the number of dispensed items reflects the frequency of prescribing and can indicate take-home dosing practices. Considering both outcomes together helps distinguish between changes in overall treatment volume and changes in take-home dosing.

For example, if DDDs increase while the number of dispensed items remains stable, this suggests higher doses per prescription, potentially indicating more take-home allocation. Conversely, if DDDs are stable or decreasing while dispensed items increase, this may reflect more frequent prescribing of smaller quantities, suggesting increased supervision. When both DDDs and dispensed items increase (or decrease), this may instead reflect growth (or reduction) of the treated population. These outcomes, derived from the EPD, allow us to assess changes in prescribing patterns and supervision practices over time.

The number of dispensed items of each preparation of methadone or buprenorphine was directly retrieved from the EPD, then summed at the practice level, and then aggregated into a total dispensed items in England. To calculate DDDs, the monthly total amount of each medication (in milligrams), derived by multiplying the strength and dispensed quantity of each methadone or buprenorphine preparation dispensed each month, was firstly summed by practices and then aggregated into a total amount of all practices in England. This total was further divided by the individual drug's standardised DDD [22], providing a metric for evaluating drug use at a population level.

Monthly changes in DDDs and dispensed items during the COVID-19 lockdown (from March 2020 to February 2021) were also calculated for each practice by subtracting monthly DDDs or dispensed items from the mean value of the three months pre-lockdown (December 2019 to February 2020). These values were standardised as percentages to adjust for the difference in baseline value and facilitate comparison across practices.

### Socioeconomic status

To evaluate the socioeconomic context, each general practice was linked to its corresponding IMD decile based on the postcode [23]. The IMD score is a measure of SES, and it was calculated by weighting the seven domains for every Lower-layer Super Output Area. The IMD decile categorises areas in England from the most deprived (decile 1, the 10% most deprived area) to the least deprived (decile 10, the 10% least deprived area).

### Data analysis

An interrupted time-series analysis was conducted on monthly DDDs and dispensed items of methadone and buprenorphine from March 2019 to February 2022. It was applied to assess the effects of COVID-19 lockdown measures on prescription OAT dispensed patterns across three periods: the pre-lockdown period (beginning in March 2019), the lockdown period (beginning in March 2020), and the post-reopening period (beginning in March 2021) [24]. The model is as follows:

$$ \begin{aligned} Y_{t} = \, \beta_{0} + \, \beta_{1} *time + \, \beta_{2} *{\mathrm{int}} ervention_{1} \\  + \, \beta_{3} *time \, after \, {\mathrm{int}} ervention_{1} \\  + \, \beta_{4} *{\mathrm{int}} ervention_{2} \\  + \, \beta_{5} *time \, after \, {\mathrm{int}} ervention_{2} \\ \end{aligned} $$

Y_*t*_: expected outcome measures in time t from March 2019 to February 2022. β_0_: intercept. *time*: 1, 2,3…t, the monthly time point from March 2019 to February 2022. *intervention*_*1*_: the launch of COVID-19 lockdown in March 2020. *intervention*_*2*_: the remove of COVID-19 lockdown in March 2021.

This model evaluated several parameters, including the baseline trend prior to lockdown (β_1_), immediate change in levels following lockdown (β_2_), change in trend following lockdown (β_3_), immediate change in level post-reopening (β_4_), and change in trend after reopening (β_5_).

Stationarity was assessed using the Dickey-Fuller test, while the Durbin-Watson test evaluated autocorrelation [25]. An autoregressive integrated moving average (ARIMA) model was further employed to address non-stationarity and autocorrelation in the data, enabling accurate estimation of changes in patterns of prescription OAT dispensed [26]. The ARIMA parameters were determined based on inspection of the autocorrelation function and partial autocorrelation plots to identify appropriate autoregressive and moving average terms [26].

To explore variations among practices in response to the lockdown, a group-based trajectory model (GBTM) was applied [27]. Only practices with consistent prescription OAT dispensed (any methadone or buprenorphine prescription dispensed from December 2019 to February 2020 and for more than 6 months during the COVID-19 lockdown period) were included, excluding those with low or sporadic prescriptions dispensed allowed the GBTM to converge better. GBTM grouped practices with similar trajectories based on the change in monthly DDDs or dispensed items during the COVID-19 lockdown (March 2020 to February 2021) by a censored normal distribution model.

A joint decision determined the final model selection after considering (1) the Bayesian information criterion value, (2) the average probability of each practice being assigned to each group was above 70%, (3) the size of each group was above 5%, and (4) the implications in clinical practices or public health. Descriptive analyses were conducted to describe the distribution of trajectory groups across IMD deciles. We performed statistical analyses using STATA-18.

## Results

### Study population

A total of 11,157 general practices in England prescribed any medication (not limited to OAT) between March 2019 and February 2022. Among these, 4273 (38%) practices with either methadone or buprenorphine prescribed and dispensed were included in this study (Supplemental Material Fig. S1).

### Impact of COVID-19 lockdown on prescriptions in England

Following the COVID-19 lockdown, there was a significant immediate increase in the monthly number of DDDs of methadone (level change, β_2_: 473,678, p = 0.002, 11.7% increase compared to the mean value in 3 months before the lockdown). However, despite this initial rise, the trend in monthly DDDs decreased significantly (β_3_: − 46,396, p = 0.03) after the COVID-19 lockdown, contrasting with the flat baseline trend prior to lockdown (β_1_: 21,885, p = 0.14). It represents a 1.2% decrease each month compared to the monthly DDDs in February 2020.

**Fig. 1:**
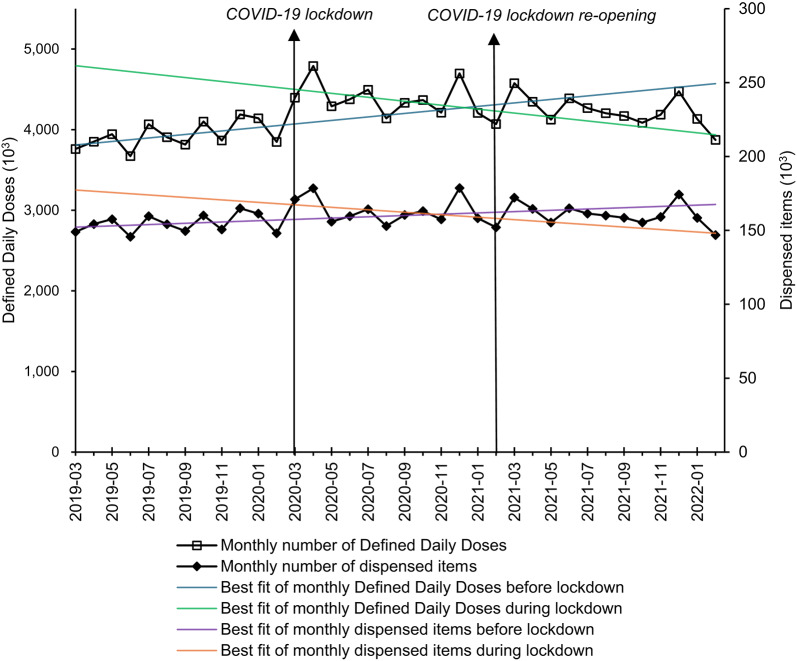
Monthly defined daily doses and dispensed items of methadone. The analysis included the 4273 practices from March 2019 to February 2022. The COVID-19 lockdown launched in March 2020 and terminated in February 2021

The monthly level of dispensed items of methadone showed no significant change following the COVID-19 lockdown (β2: 2054, p = 0.212). However, the trend in dispensed items decreased significantly (β3: − 1118, p < 0.001), contrasting with an initially rising baseline trend (β1: 1012, p < 0.001). Overall, the trend in dispensed items shifted to a significant decline (slope: − 106 items each month, 0.07% decrease each month compared to the dispensed items in February 2020) following the COVID-19 lockdown (Table 1 and Fig. 1).

**Table 1:** Parameters estimated from interrupted time series analysis

	Estimated parameters from autoregressive integrated moving average model				
	β_1_ (95%CI) Trend pre-lockdown	β_2_ (95%CI) Change in level after the start of lockdown	β_3_ (95%CI) Change in trend after the start of lockdown	β_4_ (95%CI) Change in level after the removal of lockdown	β_5_ (95%CI) Change in trend after the removal of lockdown
*Methadone*					
Monthly number of DDDs^#^	21,885 (− 7603, 51,374) p = 0.140	473,678* (184,250, 763,106) p = 0.002	− 46,396* (− 88,099, − 4693) p = 0.030	205,940 (− 83,487, 495,368) p = 0.157	− 6228 (− 47,931, 35,474) p = 0.762
Monthly number of dispensed items	1012* (676, 1348) p < 0.001	2054 (− 1170, 5279) p = 0.212	− 1118* (− 1457, − 779) p < 0.001	1926.1 (− 3827, 7679) p = 0.512	− 320 (− 832, 190) p = 0.210
*Buprenorphine*					
Monthly number of DDDs	822 (− 5052, 6698) p = 0.777	114,041* (42,375, 185,708) p = 0.002	298 (− 27,721, 28,319) p = 0.983	76,029 (− 110,744, 262,803) p = 0.425	− 5001 (− 30,591, 20,587) p = 0.702
Monthly number of dispensed items	− 6 (− 234, 222) p = 0.957	8886* (6993, 10,780) p < 0.001	− 82 (− 308, 142) p = 0.473	− 1271 (− 2909, 366) p = 0.128	55 (− 168, 278) p = 0.628

95%CI: 95% confidence interval, ^#^DDDs: Defined Daily Doses, *p < 0.05, The ARIMA parameters (p,d,q) for monthly DDDs of methadone, monthly dispensed items of methadone, monthly DDDs of buprenorphine, and monthly dispensed items of buprenorphine were (0,0,0), (0,0,3), (1,1,0), and (0,0,1), respectively.

**Fig. 2:**
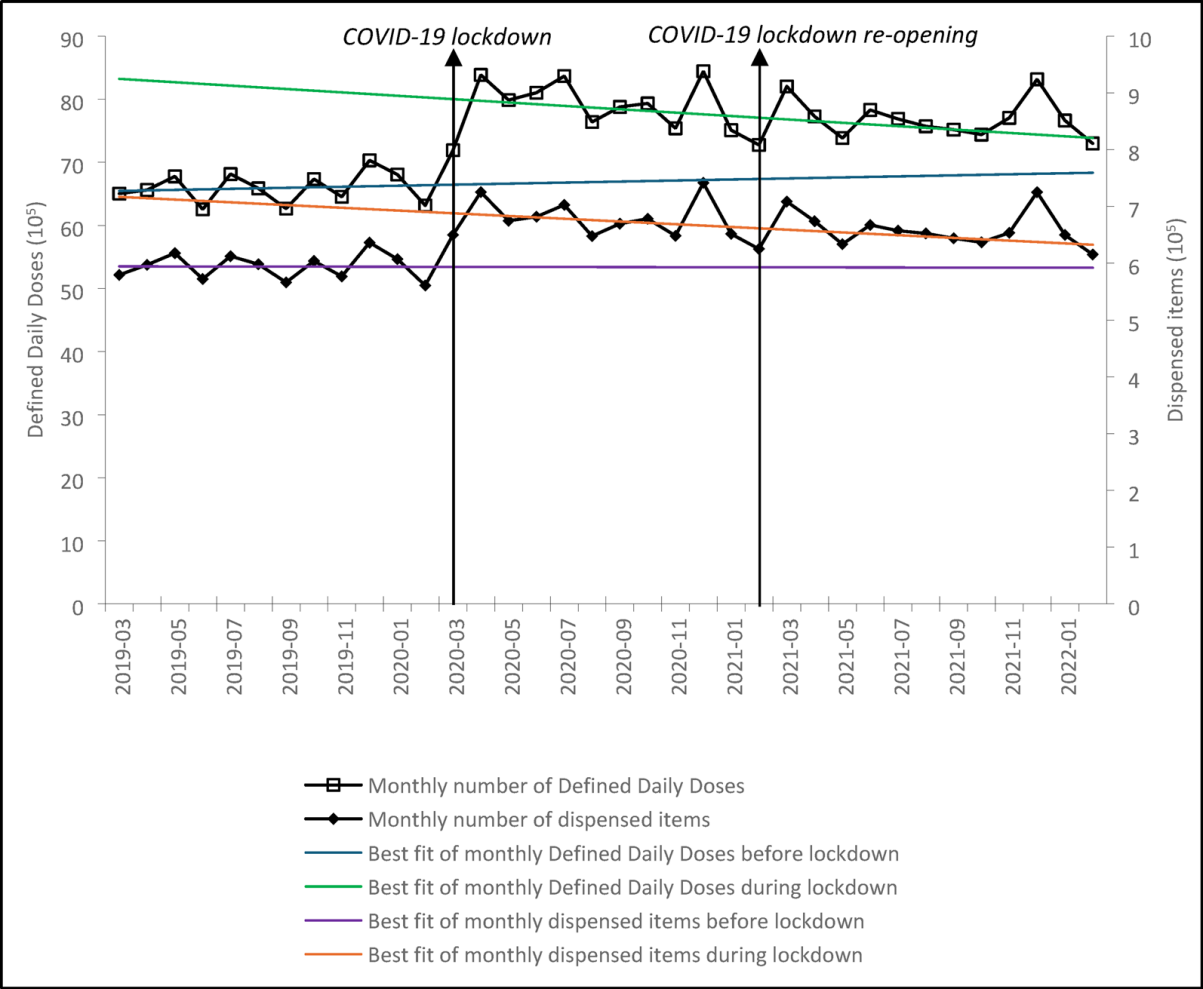
Monthly defined daily doses and dispensed items of buprenorphine. The analysis included the 4273 practices from March 2019 to February 2022. The COVID-19 lockdown launched in March 2020 and terminated in February 2021

For buprenorphine, there was also a significant immediate increase in the monthly level of DDDs following the start of lockdown (β_2_: 114,041, p = 0.002, 17% increase compared to the mean value in 3 months before the lockdown). Unlike methadone, however, the trend in the monthly DDDs of buprenorphine remained unchanged (β_3_: 298, p = 0.983), consistent with the stable baseline trend (β_1_: 822, p = 0.777). Similarly, the monthly level of dispensed buprenorphine items showed a significant immediate increase after the start of lockdown (β_2_: 8886, p < 0.001, 14.8% increase compared to the mean value in 3 months before the lockdown). Still, there was no significant change in the trend (β_3_: − 82, p = 0.473) from the steady baseline (β_1_: − 6, p = 0.957) (Table 1 and Fig. 2).

Upon the termination of the COVID-19 lockdown, no significant changes were observed in either the level (β4) or the trend (β5) when compared to the level and trend of monthly DDDs and dispensed items during lockdown for both methadone and buprenorphine, suggesting that patterns of prescription OAT dispensed stabilised post-lockdown.

### Patterns in dispensing trajectories among general practices during lockdown

Of the included 4,273 practices, 1811 (42%) practices that had methadone (n = 1200) or buprenorphine (n = 1027) prescription dispensed were included in the GBTM analysis (Supplemental Material Fig. S1). Compared to practices located in the most deprived areas (methadone: 1219 of the 2077 practices, 58.7%; buprenorphine: 966 of the 1715 practices, 56.3%), practices located in the less deprived areas (methadone: 793 of 1135 practices, 70.9%; buprenorphine: 604 of the 882 practices, 68.5%) were more likely to be excluded from the GBTM analysis due to inconsistent prescription dispensed of methadone or buprenorphine.

**Fig. 3:**
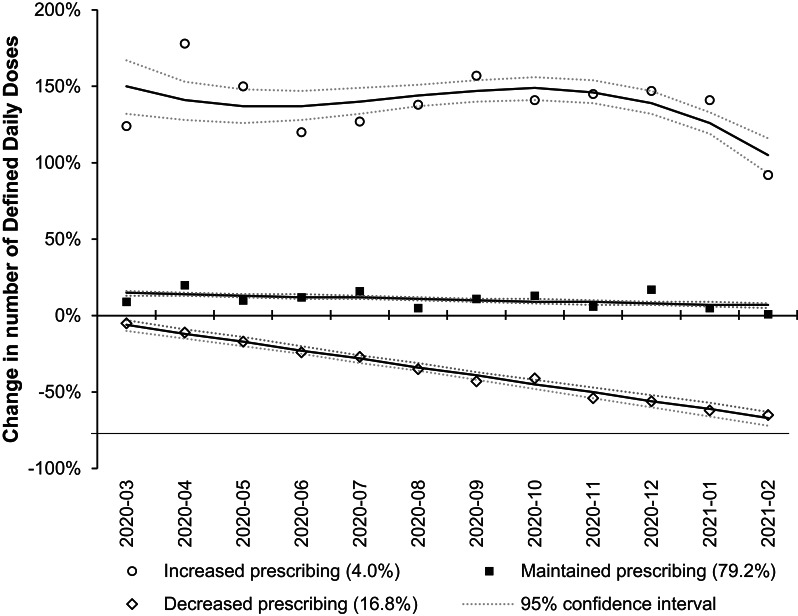
Trajectory of change in monthly defined daily doses of methadone. The analysis included the 1200 practices with consistent prescription methadone dispensed from March 2020 to February 2021

Among practices with consistent prescription OAT dispensed during the COVID-19 lockdown period, significant variation was observed in the monthly trajectories of DDDs and dispensed items for methadone and buprenorphine across general practices during the COVID-19 lockdown period. For methadone, 1,200 practices were categorised into three groups based on DDD changes: those with increasing dispensing (48 practices, 4%), stable dispensing (954 practices, 79.2%), and decreasing dispensing (198 practices, 16.8%) (Fig. 3). When grouped by changes in the number of dispensed items, the same 1,200 practices formed three similar trajectories: increasing dispensing (79 practices, 6.5%), stable dispensing (892 practices, 74.1%), and decreasing dispensing (228 practices, 19.4%) (Supplemental Material Fig. S2).

**Fig. 4:**
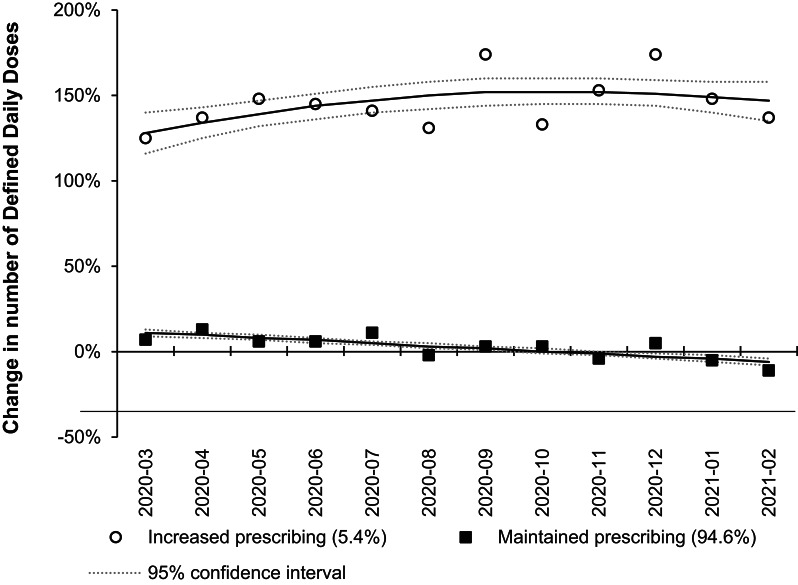
Trajectory of change in monthly Defined Daily Doses of buprenorphine. The analysis included the 1027 practices with consistent prescription buprenorphine dispensed from March 2020 to February 2021

In contrast, buprenorphine dispensing patterns showed less heterogeneity. Among the 1,027 practices included, two distinct groups emerged for both DDD and dispensed item changes. Based on monthly DDDs, practices were classified as either increasing dispensing (55 practices, 5.4%) or maintaining dispensing levels (972 practices, 94.6%) (Fig. 4). Similarly, the analysis of dispensed items resulted in groups showing increasing dispensing (51 practices, 5.0%) and stable dispensing (976 practices, 95.0%) (Supplemental Material Fig. S3). Practices with increasing methadone or buprenorphine prescription dispensed indicated around a 100–150% increase in monthly DDD compared to the mean monthly DDDs from December 2019 to February 2020.

### Factors associated with trajectory groups

**Fig. 5:**
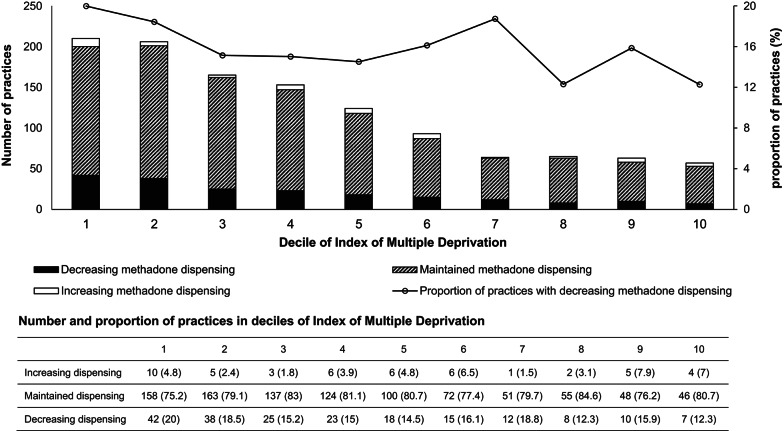
Practices in different trajectory groups of dispensing of methadone from March 2020 to February 2021 across the IMD decile. IMD: Index of Multiple Deprivation. *Note*: IMD decile 1 indicates the most deprived, and decile 10 indicates the least deprived

Among practices categorised with decreasing monthly DDDs of methadone, 141 practices (71.2%) were similarly classified into the decreasing group based on dispensed items, indicating strong concordance in declining dispensing patterns (Supplemental Material Table S2). There was a tendency for a higher proportion of practices located within deprived areas to be grouped into the trajectory of decreasing methadone dispensing (Fig. 5). In contrast, no significant difference was found in the trajectory of increased buprenorphine dispensing between practices located within areas with differing SES.

## Discussion

### Main findings

The level of monthly DDDs of methadone increased (473,678 DDDs, around 12% compared to the mean value in 3 months before the lockdown) while the dispensed items remained stable in March 2020, which implied patients receiving more doses of methadone in the short term after the launch of the COVID-19 lockdown. However, there was a decreasing trend in monthly DDDs (a 1.2% decrease each month) and dispensed items (a 0.07% decrease each month) of methadone during the COVID-19 lockdown. Those findings indicate temporal variation, a decreased trend in methadone prescriptions dispensed after an initial increase during the COVID-19 lockdown.

Around 58% of practices had inconsistent prescription OAT dispensed during the COVID-19 lockdown, especially practices located in the less deprived areas. For practices that had consistent prescription methadone dispensed during the COVID-19 lockdown, 198 practices had decreasing monthly DDDs of methadone, and around 70% of them decreased the monthly dispensed items and tended to be from a more deprived area where practices were allocated with similar resources, but the patients had a higher rate of drug-related deaths and often with more complex health issues [16, 17, 28].

In contrast to methadone, the level of monthly DDDs (114,041 DDDs, 17% increase) and dispensed items (8886 items, 14.8% increase) of buprenorphine both increased in March 2020. The stable trends during the COVID-19 lockdown indicated that more patients received and maintained buprenorphine therapy during the lockdown period. In contrast to methadone, prescription buprenorphine dispensed was not related to the SES of areas where practices were located. These findings suggest that socioeconomic deprivation may be a potential limiting factor in expanding methadone dispensing during the pandemic, while buprenorphine dispensing appears less impacted by SES.

### Comparison with existing literature

Similar to a published study in Scotland, which documented the declining trend in the dispensing of methadone [20], our study observed a similar decline and additionally found an immediate increase in the level of monthly DDDs of methadone in March 2020. The immediate elevation might imply an increase in the average quantity prescribed per methadone prescription, a phenomenon that has also been reported in the United States (US) and Scotland [20, 29].

A published study indicated concerns about a higher dropout rate in Ukraine during the COVID-19 pandemic [30]. Although our study did not use individual patient data, we observed a decreasing trend in the dispensed items and DDDs of methadone, which may reflect a reduction in the number of patients remaining in treatment, consistent with an increase in dropout. This also highlights the importance of supervised consumption for methadone users to stay in the program [31].

In contrast, there was no change in the trend of dispensed buprenorphine items in our study, indicating no change in dropout but an increase in the number of individuals treated with buprenorphine after the lockdown was implemented. Similarly, a study conducted in Ireland found a decreasing dropout rate among buprenorphine users [32].

However, unlike the publication that adopted data from selected regions in Scotland [20], our study indicated a significant increase in the dispensing of buprenorphine when including all prescription OAT dispensed in England. The discrepancy may be attributed to the use of different outcome measures and a nationwide database in our analysis. A study evaluated the impact of lockdown on the prescription opioid analgesics dispensed and observed a non-significant change in the level and trend in England [33], which might imply the limited impacts of lockdown on most prescribed medications for which supervised consumption is not required.

A cross-sectional study in the US identified a variation in trends of prescription methadone dispensed across states during the COVID-19 pandemic [29]. Our study contributes to the understanding of such variations at the practice level in England, highlighting their potential associations with SES. According to the Local Government Association, deprived SES, including limited income, insecure employment, no public transportation, and overcrowded housing, could be a barrier to access to healthcare during the COVID-19 lockdown [34]. Unscheduled admissions rates fell more in the most deprived than the least deprived areas in England, Scotland and Wales [35]. In addition, patients from deprived areas have multiple comorbidities, which put them at a higher risk of COVID-19 infection, and hence make them susceptible to self-isolation [36].

### Implications for research

There was a higher prevalence of OUD in more deprived areas [37], and increasing methadone-related mortality was observed during the COVID-19 pandemic in England, especially among patients without established prescribing records [38–40]. Treatment dropout is associated with increased mortality risk among patients receiving methadone [3]. Therefore, the practice guidance should emphasise the importance of preventing treatment dropout and enhancing patient education on methadone overdose prevention.

This study indicated that half of the practices had prescription methadone dispensed for less than six months during the COVID-19 lockdown, and a higher proportion of practices located in deprived areas reduced methadone dispensing. This suggests there might be variation in the implementation of the 'take-home doses' policy, and in addition to OAT access, an additional intervention to prevent harm might be needed for OUD patients who most require it.

There were several reasons to facilitate or hinder the implementation of the 'take-home doses' policy, such as ongoing substance use disorders, the ability to store methadone securely, and the involvement of multidisciplinary teams to determine appropriate dosing [18]. These insights underscore the necessity for targeted interventions, such as housing and psychological support and online peer support groups, to address the challenges in accessing OAT in deprived SES contexts, particularly during public health emergencies.

Our study found an increase in prescription buprenorphine dispensed, which may reflect a substitution of methadone. However, the applied data sources did not allow for the identification of patients who transitioned from methadone to buprenorphine. Further studies applying individual patient data are warranted to identify the switching of OAT in response to public health challenges and investigate the safety of switching from methadone to buprenorphine.

### Strengths and limitations

This study used prescription data from all general practices which prescribed OAT in England to evaluate the impact of the COVID-19 lockdown, ensuring that the results were highly representative. By extending the analysis to February 2022, our study can effectively quantify and evaluate the prescriptions dispensed during the COVID-19 lockdown and after COVID-19 reopening. To minimize quantification errors, we employed the BNF codes to include methadone and buprenorphine preparations for opioid dependence instead of pain relief.

There were some limitations in this study. The interrupted time-series analysis can only evaluate the impact of a single event, and hence this study cannot fully account for the impact of concurrent events during the same period, such as medication shortages. In addition, practices in England were grouped into clinical commissioning groups (CCGs), which are responsible for the commissioning of healthcare services in a local area. Still, CCGs were restructured in April 2020, and our study is not able to quantify the influence [41].

Additionally, GBTMs are mainly descriptive analyses, and hence we cannot rule out the impacts of other events prior to or during the COVID-19 lockdown. Also, this study was unable to identify the association between trajectory groups and characteristics of registrants, such as age and gender, because most included practices lacked comprehensive registrant data. Nevertheless, we incorporated SES of areas where practices are located in the analysis, as it is often associated with the characteristics of registrants [42, 43].

To describe the long-term trajectory, this study included only practices with consistent prescription OAT dispensed, which might potentially bias the results. However, our preliminary works showed that over 95% of prescription OAT dispensed in UK primary care was from the top 20% of practices (Supplemental Material Result S1), and hence the influence of excluding practices with sporadically prescription OAT dispensed should be limited. Moreover, although primary care serves as the gatekeepers in England, patients could be referred to secondary care during the COVID-19 lockdown, and we can only quantify the prescription OAT dispensed in primary care.

Lastly, this study applied aggregate-level data, which does not provide the prescribing dose from individual patients and hence may mask variability in treatment approaches within practices. The results should be interpreted with caution. Further studies should explore the nuances of prescribing behaviours and their associated factors, particularly in the context of changing healthcare policies and socioeconomic conditions.

## Conclusions

The dispensing of methadone increased temporarily in March 2020. Still, there was variation in the implementation of the 'take-home doses' policy, and practices in more deprived areas have a tendency to reduce methadone dispensing during the COVID-19 lockdown. The dispensing of buprenorphine increased and remained stable after the launch of the COVID-19 lockdown. Further studies should evaluate the short and long-term effectiveness and mortality of patients switching from methadone to buprenorphine or retention in treatment during the lockdown.

## Supplementary Information

Below is the link to the electronic supplementary material.

Supplementary Material 1.

## Data Availability

This study used publicly available data from the National Health Service Business Services Authority.

## Abbreviations

OUD: Opioid use disorder
OAT: Opioid agonist therapy
SES: Socioeconomic status
DDDs: Defined daily doses
COVID-19: Coronavirus disease 2019
IMD: Index of multiple deprivation
BNF: British National Formulary
GBTM: Group-based trajectory model
UK: United Kingdom
