# Correction: Impact of COVID-19 lockdown on methadone and buprenorphine prescriptions in England primary cares: an interrupted time series analysis

**DOI:** 10.1186/s12954-026-01438-6

**Published:** 2026-04-16

**Authors:** Yi-Chen Chang, Wan-Chuen Liao, Li-Chia Chen, Teng-Chou Chen

**Affiliations:** 1 Department of Pharmacy, National Yang-Ming Chiao-Tung University, Number 155, Section 2 Linong Street, Beitou District, Taipei City, 112304 Taiwan; 2 Centre for Pharmacoepidemiology and Drug Safety, Division of Pharmacy and Optometry, The University of Manchester, 1st Floor, Stopford Building, Oxford Road, Manchester, M13 9PT UK; 3 School of Dentistry, College of Medicine, National Taiwan University, Number 1, Changde Street, Zhongzheng District, Taipei City, 100 Taiwan

**Correction to: Harm Reduction Journal (2025) 22:203** 10.1186/s12954-025-01354-1

In this article [1], Figures 1 and 2 look the same in the published paper. For completeness and transparency, the incorrect and correct versions of Fig. 2 are displayed below. The original article has been corrected.

Incorrect Fig. 2:

**Fig. 2:**
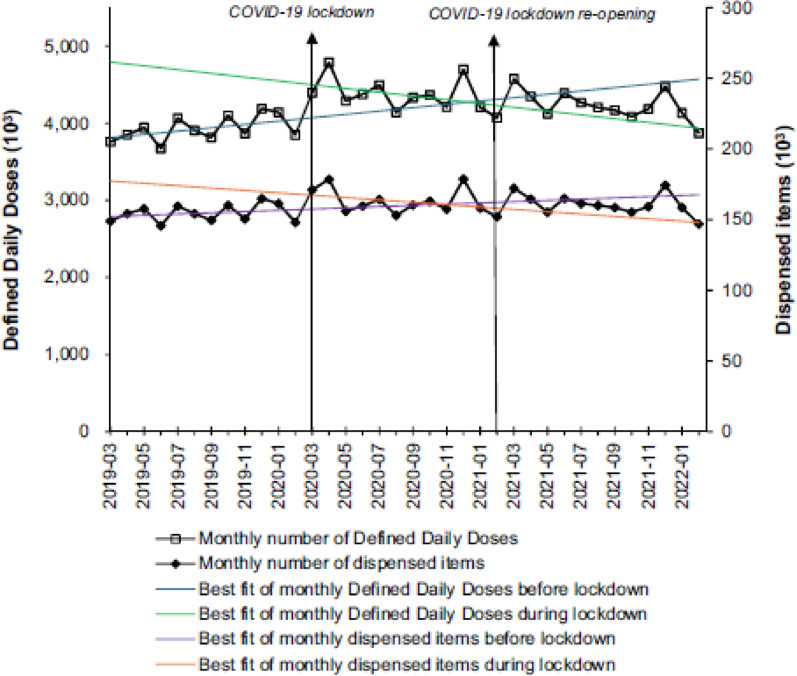
Monthly defined daily doses and dispensed items of buprenorphine. The analysis included the 4273 practices from March 2019 to February 2022. The COVID-19 lockdown launched in March 2020 and terminated in February 2021

Correct Fig. 2:

**Fig. 2:**
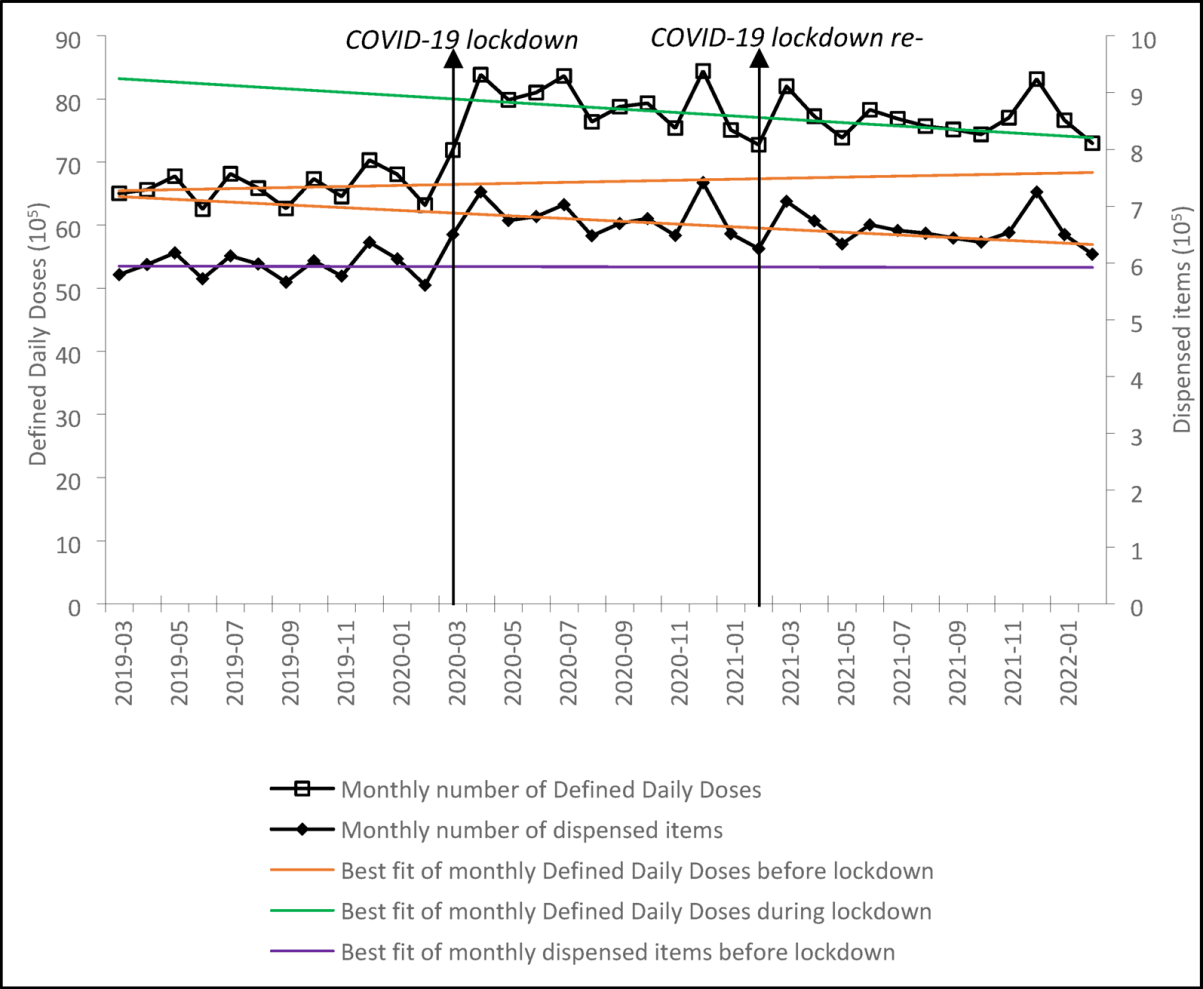
Monthly Defined Daily Doses and dispensed items of buprenorphine. The analysis included the 4,273 practices from March 2019 to February 2022. The COVID-19 lockdown launched in March 2020 and terminated in February 2021
