# Supplementary material for: Impact of COVID-19 lockdown on methadone and buprenorphine prescriptions in England primary cares: an interrupted time series analysis
Source: Harm Reduct J. 2025 Nov 25;22:203. doi: 10.1186/s12954-025-01354-1 (PMC12750698; doi:10.1186/s12954-025-01354-1)
Supplement: Supplementary file 1 — Supplementary Material 1. [file 12954_2025_1354_MOESM1_ESM.docx]

**Supplemental Materials**

Supplemental Material Table S1 The prescribing recommendation of methadone and buprenorphine before and during COVID-19 lockdown

|  | Before COVID-19 lockdown | During COVID-19 lockdown |
| --- | --- | --- |
| Methadone | Prescribing with supervision was recommended from treatment initiation until the patient achieves stability. | Methadone can be prescribed for one to two weeks' take-home doses', and patients were allowed to be administered without supervision |
| Buprenorphine | Prescribing with unsupervised take-away doses. | Prescribing with unsupervised take-away doses. |

Figure S1. Flowchart indicating the number of included general practices. 4,273 practices were included to evaluate the impact of COVID-19 lockdown policy. 1,811 practices were included to explore the variation in prescribing during COVID-19 lockdown. GPs: general practices.

11,157 GPs which prescribed any drugs during March 2019 to February 2022

4,273 GPs which prescribed methadone or buprenorphine from March 2019 to February 2022 (to evaluate the impact of COVID-19)

- 6,884 GPs which did not prescribe methadone and buprenorphine during March 2019 to February 2022

1,811 included GPs

(1,200 GPs which prescribed methadone, 1,027 GPs prescribed buprenorphine)

(to explore the variation across GPs)

- 2,525 GPs which did not prescribed methadone and buprenorphine from December 2019 to February 2020.
- 506 GPs which had prescribed methadone and buprenorphine less than 6 months from March 2020 to February 2021

Figure S2. Trajectory of change in monthly methadone dispensed items from March 2020 to February 2021.

Figure S3. Trajectory of change in monthly buprenorphine dispensed items from March 2020 to February 2021.

Table S2. General practices in trajectory groups of change in methadone utilisation and dispensed items.

|  |  | Trajectory group of dispensed items | | |  |
| --- | --- | --- | --- | --- | --- |
|  |  | Decreased | Maintained | Increased | Total |
| Trajectory group of DDDs* | Decreased | 141 | 55 | 2 | 198 |
|  | Maintained | 86 | 822 | 46 | 954 |
|  | Increased | 1 | 15 | 31 | 47 |
|  | Total | 228 | 892 | 79 | 1,199 |
| *DDDs: Defined daily doses | | | | | |

Result S1. Results from a preliminary study exploring the dispensing of prescribed methadone and buprenorphine from December 2019 to February 2020.

The preliminary study included 1748 practices that prescribed any methadone from December 2019 to February 2020. For each practice, we calculated the mean monthly Defined Daily Doses (DDDs) between December 2019 and February 2020 and categorised those practices into deciles based on the mean monthly DDDs of prescribed methadone. For each decile, the total prescribed amount of methadone was summed. Overall, there were 175 practices in the top decile, and those practices prescribed 89.3% of methadone in England. Practice in the second decile prescribed 5.5% of methadone in England.

Similarly, 1452 practices prescribed any buprenorphine from December 2019 to February 2020 and were categorised into deciles. Practices in the top decile prescribed 84.4% of buprenorphine in England and 91.8% of the buprenorphine were prescribed by the top 20% of the practices.
